# Internalin profiling and multilocus sequence typing suggest four *Listeria innocua *subgroups with different evolutionary distances from *Listeria monocytogenes*

**DOI:** 10.1186/1471-2180-10-97

**Published:** 2010-03-31

**Authors:** Jianshun Chen, Qiaomiao Chen, Lingli Jiang, Changyong Cheng, Fan Bai, Jun Wang, Fan Mo, Weihuan Fang

**Affiliations:** 1 Zhejiang University Institute of Preventive Veterinary Medicine, and Zhejiang Provincial Key Laboratory of Prevent Veterinary Medicine, Hangzhou, PR China; 2 Zhejiang California International NanoSystems Institute, Hangzhou, PR China

## Abstract

**Background:**

Ecological, biochemical and genetic resemblance as well as clear differences of virulence between *L. monocytogenes *and *L. innocua *make this bacterial clade attractive as a model to examine evolution of pathogenicity. This study was attempted to examine the population structure of *L. innocua *and the microevolution in the *L. innocua*-*L. monocytogenes *clade via profiling of 37 internalin genes and multilocus sequence typing based on the sequences of 9 unlinked genes *gyrB*, *sigB*, *dapE*, *hisJ*, *ribC*, *purM*, *gap*, *tuf *and *betL*.

**Results:**

*L. innocua *was genetically monophyletic compared to *L. monocytogenes*, and comprised four subgroups. Subgroups A and B correlated with internalin types 1 and 3 (except the strain 0063 belonging to subgroup C) and internalin types 2 and 4 respectively. The majority of *L. innocua *strains belonged to these two subgroups. Subgroup A harbored a whole set of *L. monocytogenes*-*L. innocua *common and *L. innocua*-specific internalin genes, and displayed higher recombination rates than those of subgroup B, including the relative frequency of occurrence of recombination versus mutation (ρ/θ) and the relative effect of recombination versus point mutation (r/m). Subgroup A also exhibited a significantly smaller exterior/interior branch length ratio than expected under the coalescent model, suggesting a recent expansion of its population size. The phylogram based on the analysis with correction for recombination revealed that the time to the most recent common ancestor (TMRCA) of *L. innocua *subgroups A and B were similar. Additionally, subgroup D, which correlated with internalin type 5, branched off from the other three subgroups. All *L. innocua *strains lacked seventeen virulence genes found in *L. monocytogenes *(except for the subgroup D strain L43 harboring *inlJ *and two subgroup B strains bearing *bsh*) and were nonpathogenic to mice.

**Conclusions:**

*L. innocua *represents a young species descending from *L. monocytogenes *and comprises four subgroups: two major subgroups A and B, and one atypical subgroup D serving as a link between *L. monocytogenes *and *L. innocua *in the evolutionary chain. Although subgroups A and B appeared at approximately the same time, subgroup A seems to have experienced a recent expansion of the population size with higher recombination frequency and effect than those of subgroup B, and might represent the possible evolutionary direction towards adaptation to enviroments. The evolutionary history in the *L. monocytogenes*-*L. innocua *clade represents a rare example of evolution towards reduced virulence of pathogens.

## Background

The genus *Listeria *encompasses six species, which could be divided into three major phylogenetic clusters: *L. monocytogenes*-*L. innocua*, *L. ivanovii-L. seeligeri-L. welshimeri*, and *L. grayi *[1]. *L. monocytogenes *is a well-recognized intracellular pathogen of humans and animals, with clinical features including meningitis, meningoencephalitis, septicemia, abortion, perinatal infections and gastroenteritis [2]. Three genetic lineages have been identified within *L. monocytogenes *strains, with lineage I including serovars 1/2b, 3b, 4b, 4d, 4e, 4ab and 7, lineage II covering serovars 1/2a, 3a, 1/2c and 3c, and lineage III comprising sublineages IIIA, IIIB and IIIC covering serovars 4a, 4c and atypical 4b [3-5]. Of the 13 *L. monocytogenes *serovars, four (4b, 1/2a, 1/2b and 1/2c) are responsible for over 98% of human listeriosis cases whereas other serovars (e.g., 4a and 4c) are seldom implicated in listeriosis [6,7]. *L. innocua *is of particular significance because it is most closely related to *L. monocytogenes*, and they usually co-exist in various environmental, food and clinical specimens [8]. Although these two species resemble each other ecologically, biochemically and genetically, *L. innocua *has no pathogenic inclination [1,9]. Therefore, the *L. innocua-L. monocytogenes *clade within the genus *Listeria *can be used as a model system to examine the evolution of pathogenicity.

Intriguingly, some *L. monocytogenes *strains tend to lose virulence factors that play critical roles in infection, which has been considered as a rare example of evolution towards reduced virulence of pathogens [4,10]. Certain *L. monocytogenes *lineage IIIA strains are presumed to have identifiable linkage between *L. monocytogenes *and *L. innocua *by possessing many genes common to *L. monocytogenes *[e.g., *Listeria *pathogenicity island I (LIPI-1), *inlAB *locus, *bsh *and *hpt*], and sharing many gene deletions similar to *L. innocua *(e.g., *inlC*, *inlI*, *inlJ*, internalin cluster between *ascB *and *dapE*, and arginine deiminase island *lmo0036-lmo0041*) [11-13]. Therefore, the population structure and biodiversity in *L. innocua *may, from the other side of the coin, provide clues for the evolutionary history in the *L. monocytogenes*-*L. innocua *clade. Unfortunately, comprehensive knowledge on the phylogenetic structure of *L. innocua *is still lacking.

Various strain typing methods have been developed and improved with a general shift from phenotype-based to genotype-based strategies [14]. Given its accuracy, reproducibility and increased speed of DNA sequencing, DNA sequence-based multilocus sequence typing (MLST) has gained more popularity [15], and provided an overview of the population structure of *L. monocytogenes *[4,16]. Internalin profiling seems to be instrumental to subtype *L. monocytogenes *strains into different serovars [17]. Moreover, internalin loci are also present in non-pathogenic species, including *L. innocua*, and seem to play broad roles not merely limited to attachment and invasion of host cells [18-20].

In this study, we attempted to delineate a phylogenetic framework based on internalin profiling and MLST analysis of a collection of *L. innocua *isolates from various food sources, and further to investigate microevolution in the *L. innocua*-*L. monocytogenes *clade.

## Results

### Biochemical patterns of *L. innocua *and *L. monocytogenes *strains

All *L. innocua *and *L. monocytogenes *strains displayed similar utilization patterns for xylose (negative), mannitol (negative) and glucose (positive), while hemolysis could distinguish these two species with *L. monocytogenes *showing β-hemolysis and all *L. innocua *strains being non-hemolytic. With regard to the rhamnose utilization pattern, three *L. innocua *strains 386, L19 and L103 (3/34, 8.8%) as well as *L. monocytogenes *sublineages IIIB and IIIC strains covering serovars 4a, 4b and 4c were atypically negative for rhamnose fermentation (Table 1).

**Table 1:** MLST and mouse assay of *L. innocua *and *L. monocytogenes *strains

Strain			Source	Subgroup/ lineage (serovar)	rhamnose	Allele designation										Virulence gene		Mouse assay	
				
						*gyrB*	*dapE*	*hisJ*	*sigB*	*ribC*	*purM*	*betL*	*gap*	*tuf*	ST^a^	*bsh*	*inlJ*	CFU	Relative virulence (%)
*L. innocua*																			
ATCC33090			reference	A	+	1	1	1	1	1	1	1	1	1	1	--	--	3.5 × 10^7^	0
90001			reference	B	+	2	2	2	2	2	2	1	1	2	2	--	--	2.7 × 10^7^	0
1603			reference	B	+	3	3	3	3	3	3	2	1	2	3	+	--	2.0 × 10^7^	0
AB2497			reference	A	+	1	1	4	1	1	1	1	1	1	4	--	--	3.3 × 10^7^	0
CLIP11262			reference	A	+	1	4	5	1	1	4	3	1	1	5	--	--	ND	ND
0063			meat	C	+	4	5	6	4	4	5	4	1	1	6	--	--	5.3 × 10^7^	0
0065			meat	A	+	1	6	5	1	1	4	3	1	1	7	--	--	1.5 × 10^7^	0
0068			meat	B	+	2	2	5	2	5	2	5	1	2	8	--	--	1.8 × 10^7^	0
0072			meat	A	+	1	7	5	5	1	4	3	2	1	9	--	--	2.1 × 10^7^	0
0082			meat	A	+	1	7	5	1	1	4	3	1	1	10	--	--	2.3 × 10^7^	0
0083			meat	A	+	1	8	5	1	1	4	3	1	1	11	--	--	1.7 × 10^7^	0
0173			meat	A	+	4	9	1	6	6	1	6	1	1	12	--	--	2.2 × 10^7^	0
0197			meat	A	+	4	10	1	6	6	1	6	1	1	13	--	--	1.9 × 10^7^	0
01174			meat	A	+	5	11	7	7	7	4	1	1	1	14	--	--	1.3 × 10^7^	0
01178			meat	A	+	1	12	5	1	1	4	3	1	1	15	--	--	3.3 × 10^7^	0
01182			meat	A	+	1	13	5	1	1	4	3	1	1	16	--	--	2.3 × 10^7^	0
317			milk	A	+	1	1	5	1	1	1	7	1	1	17	--	--	3.3 × 10^7^	0
337			milk	B	+	3	3	3	3	3	3	2	1	2	3	--	--	2.0 × 10^7^	0
376			milk	A	+	1	1	1	1	1	1	1	1	1	1	--	--	2.5 × 10^7^	0
380			milk	B	+	3	3	3	3	8	3	2	1	2	18	--	--	2.1 × 10^7^	0
386			milk	B	--	5	14	8	8	9	4	8	1	1	19	+	--	3.0 × 10^7^	0
438			milk	B	+	6	15	9	9	10	6	3	3	1	20	--	--	1.6 × 10^7^	0
693			milk	A	+	1	12	7	1	1	4	3	1	1	21	--	--	2.3 × 10^7^	0
694			milk	A	+	5	11	1	7	11	4	9	1	1	22	--	--	4.7 × 10^7^	0
ZS14			seafood	A	+	1	12	5	1	1	7	10	1	1	23	--	--	4.3 × 10^7^	0
ZXF			seafood	B	+	3	3	3	3	12	3	2	1	2	24	--	--	3.3 × 10^7^	0
1571			seafood	A	+	1	16	5	1	1	4	3	1	1	25	--	--	2.0 × 10^7^	0
L19			seafood	B	--	7	17	10	10	13	8	11	4	2	26	--	--	1.5 × 10^7^	0
L43			seafood	D	+	8	18	11	11	14	9	12	5	3	27	--	+	1.7 × 10^7^	0
NB2			seafood	B	+	3	3	3	3	15	3	2	1	2	28	--	--	3.5 × 10^7^	0
NB3			seafood	B	+	3	3	3	3	15	3	2	1	2	28	--	--	3.7 × 10^7^	0
NB24			seafood	B	+	5	19	12	12	16	2	13	1	1	29	--	--	2.9 × 10^7^	0
L87			pork	B	+	5	19	12	7	16	10	13	1	1	30	--	--	1.3 × 10^7^	0
L103			chicken	A	--	1	12	5	1	1	11	14	1	1	31	--	--	4.0 × 10^7^	0
*L. monocytogenes*																			
SH3			pork	I (1/2b)	+	9	20	13	13	17	12	15	6	4	32	+	+	4.3 × 10^7^	100
NB26			seafood	I (1/2b)	+	10	21	14	14	18	13	16	6	4	33	+	+	6.5 × 10^7^	100
NB27			seafood	I (1/2b)	+	11	22	15	14	19	14	17	7	4	34	+	+	5.5 × 10^7^	80
M1			milk	I (1/2b)	+	11	23	13	14	19	15	18	8	4	35	+	+	3.0 × 10^7^	100
ScottA			reference	I (4b)	+	11	24	15	15	19	16	19	8	4	36	+	+	3.3 × 10^7^	100
NB4			seafood	I (4b)	+	11	25	16	15	19	14	20	6	4	37	+	+	2.1 × 10^7^	100
NB6			seafood	I (4b)	+	11	26	17	16	20	16	21	6	4	38	+	+	3.3 × 10^7^	100
NB7			seafood	I (4b)	+	11	26	17	16	20	16	22	6	4	39	+	+	4.6 × 10^7^	100
NB25			seafood	I (4b)	+	11	27	18	15	19	17	19	6	4	40	+	+	4.6 × 10^7^	100
90SB1			animal	I (4b)	+	11	27	16	15	19	17	19	6	4	41	+	+	5.5 × 10^7^	100
EGDe			reference	II (1/2a)	+	12	28	19	17	21	18	20	9	5	42	+	+	5.5 × 10^7^	100
10403S			reference	II (1/2a)	+	13	29	20	17	22	19	20	10	5	43	+	+	5.0 × 10^7^	100
SH2			vegetable	II (1/2a)	+	13	29	21	17	23	20	20	9	5	44	+	+	4.3 × 10^7^	100
SH4			chicken	II (1/2a)	+	13	29	20	17	22	19	20	11	5	45	+	+	5.0 × 10^7^	100
NB5			seafood	II (1/2a)	+	13	29	22	17	24	21	23	12	5	46	+	+	4.5 × 10^7^	100
NB21			seafood	II (1/2a)	+	13	29	23	17	25	22	23	13	5	47	+	+	3.9 × 10^7^	80
P3			pork	II (1/2a)	+	13	23	24	18	26	23	24	13	5	48	+	+	4.0 × 10^7^	100
NB28			seafood	II (1/2c)	+	12	23	19	17	21	18	20	14	6	49	+	+	4.1 × 10^7^	100
V1			vegetable	II (1/2c)	+	12	23	19	17	21	18	20	9	6	50	+	+	3.0 × 10^7^	100
P19			chicken	II (1/2c)	+	12	30	19	17	21	18	20	9	6	51	+	+	5.0 × 10^7^	100
54006			reference	IIIA (4a)	+	14	31	25	19	27	24	3	15	2	52	+	--	1.3 × 10^7^	0
F2-695			reference	IIIA(4a)	+	15	32	26	20	28	25	25	15	7	53	+	+	1.2 × 10^7^	40
F2-086			reference	IIIB (4a)	--	16	33	27	21	29	26	26	16	8	54	+	--	1.7 × 10^7^	100
F2-407			reference	IIIB (4a)	--	17	34	28	21	30	26	27	16	9	55	+	--	1.5 × 10^7^	100
F2-270			reference	IIIB (4a)	--	18	35	29	21	31	27	28	16	8	56	+	--	2.2 × 10^7^	100
F2-208			reference	IIIC (4a)	--	19	36	30	22	32	28	29	16	10	57	+	--	3.5 × 10^7^	100
F2-525			reference	IIIA (4b)	+	20	37	31	23	33	29	30	17	11	58	+	+	2.8 × 10^7^	100
J1-158			reference	IIIB (4b)	--	21	34	28	21	29	30	31	16	8	59	+	--	2.2 × 10^7^	40
J2-071			reference	IIIA (4c)	+	22	38	26	20	28	31	25	15	12	60	+	+	1.5 × 10^7^	100
W1-111			reference	IIIC (4c)	--	23	39	32	24	34	32	32	18	2	61	+	--	2.8 × 10^7^	80
*L. welshimeri*																			
SLCC5334			reference	ND	--	24	40	33	25	35	33	33	19	13	62	--	--	ND	ND
C15			reference	ND	--	25	41	34	26	36	34	34	20	14	63	--	--	5.5 × 10^7^	0
AB1626			reference	ND	--	25	41	35	27	36	34	34	20	14	64	--	--	ND	ND
AB2500			reference	ND	--	24	40	36	25	35	33	33	19	13	65	--	--	ND	ND

ND, not done. ^a ^ST, sequence type.

### Internalin profiling indicates five internalin types (ITs) of *L. innocua*

Upon examination of 14 *L. monocytogenes*-*L. innocua *common, 4 *L. innocua*-specific and 19 *L. monocytogenes*-specific internalin genes, *L. innocua *strains harbored 15 to 18 internalin genes, with three *L. monocytogenes*-*L. innocua *common and one *L. innocua*-specific internalin genes absent individually or in combination in certain *L. innocua *strains (Table S1; Additional file 1). Eighteen *L. monocytogenes*-specific internalin genes were absent in all *L. innocua *strains except for L43 having *inlJ *(Table 1). These *L. innocua *strains could be separated into five internalin types (ITs), with IT1 containing a whole set of *L. monocytogenes*-*L. innocua *common and *L. innocua*-specific internalin genes, IT2 lacking *lin1204*, IT3 lacking *lin1204 *and *lin2539*, IT4 lacking *lin0661*, *lin0354 *and *lin2539*, and IT5 lacking *lin1204 *but bearing *inlJ*. The majority of *L. innocua *strains fell into IT1 (17/34, 50%) and IT2 (12/34, 35.4%). Among the remainders, three belonged to IT3 (8.8%), one to IT4 (2.9%) and one to IT5 (2.9%). In addition, all *L. monocytogenes *strains contained *L. monocytogenes*-*L. innocua *common internalin genes ranging from 6 to 13, and lacked all *L. innocua*-specific internalin genes (Table 2).

**Table 2:** Internalin profiling of *L. innocua *and *L. monocytogenes *strains

IT	No. of internalin genes		Characteristics	No. (%) of strains	No. (%) of strains belonging to each subgroup			
				
	common and *L. innocua* -specific	*L. monocytogenes* -specific			A	B	C	D
1	18	0	whole set of common and *L. innocua*-specific internalin genes	17 (50.0%)	17	0	0	0
2	17	0	*lin1204 *negative	12 (35.4%)	0	12	0	0
3	16	0	*Lin1204, lin2539 *negative	3 (8.8%)	2	0	1	0
4	15	0	*lin0661, lin0354, lin2539 *negative	1 (2.9%)	0	1	0	0
5	17	1	*lin1204 *negative, and *inlJ *positive	1 (2.9%)	0	0	0	1
Total	18	19	--	34	19 (55.9%)	13 (38.3%)	1 (2.9%)	1 (2.9%)

### MLST correlates with internalin profiling of *L. innocus *strains

Sixty-four strains in the *L. monocytogenes*-*L. innocua *clade were classified into 61 unique sequence types (ST) in the MLST scheme with a high discrimination index (DI = 0.99, 0.76 to 0.98 per gene). The concatenated sequence data showed that *L. innocua *was genetically monophyletic as compared to *L. monocytogenes*, with 34 *L. innocua *and 30 *L. monocytogenes *strains bearing 391 (6.69%) and 820 (14.03%) polymorphisms respectively. The average nucleotide diversity π of *L. innocua *was lower than that of *L. monocytogenes *(1.06% vs 4.38%). However, the nonsynonymous/synonymous mutation rate of *L. innocua *was higher than that of *L. monocytogenes *(0.0865 vs 0.0500) (Table 3).

**Table 3:** Polymorphisms at nine genes in the *L. innocua*-*L. monocytogenes *clade

Gene	No. strains	Size (bp)	No. alleles	No. (%) polymorphic sites	D.I.	Ks	Ka	Ka/Ks	π
*gyrB*	64	657	23	74 (11.26%)	0.91	0.1991	0.0010	0.0050	0.0384
*dapE*	64	669	39	146 (21.82%)	0.98	0.2337	0.0152	0.0650	0.0564
*hisJ*	64	714	32	187 (26.19%)	0.95	0.3999	0.0380	0.0951	0.1000
*sigB*	64	642	24	83 (12.93%)	0.92	0.2588	0.0054	0.0207	0.0518
*ribC*	64	633	34	163 (25.75%)	0.93	0.6002	0.0209	0.0348	0.1093
*purM*	64	693	31	170 (24.53%)	0.94	0.3955	0.0194	0.0490	0.0853
*betL*	64	534	32	171 (32.02%)	0.94	0.7918	0.0312	0.0394	0.1325
*gap*	64	621	18	28 (4.51%)	0.76	0.0240	0.0013	0.0547	0.0067
*tuf*	64	681	11	14 (2.06%)	0.80	0.0182	0.0021	0.1160	0.0058
Concatenated	64	5,844	61	1036 (17.73%)	0.99	0.2621	0.0147	0.0559	0.0623
Concatenated, *L. innocua*	34	5,844	31	391 (6.69%)	0.99	0.0365	0.0032	0.0865	0.0106
Concatenated, subgroupA	19	5,844	18	90 (1.54%)	0.99	0.0142	0.0018	0.1241	0.0046
Concatenated, subgroup B	13	5,844	11	135 (2.31%)	0.97	0.0280	0.0018	0.0628	0.0077
Concatenated, subgroup C	1	5,844	1	--	--	0.4659	0.0241	0.0517	--
Concatenated, subgroup D	1	5,844	1	--	--	0.4867	0.0225	0.0461	--
Concatenated, *L. monocytogenes*	30	5,844	30	820 (14.03%)	1.00	0.1781	0.0089	0.0500	0.0438
Concatenated, lineage I	10	5,844	4	84 (1.44%)	1.00	0.0174	0.0019	0.1112	0.0055
Concatenated, lineage II	10	5,844	6	250 (4.28%)	1.00	0.0493	0.0027	0.0537	0.0131
Concatenated, lineage III	10	5,844	10	522 (8.93%)	1.00	0.1459	0.0084	0.0575	0.0374

D.I.: discrimination index; Ks: number of synonymous changes per synonymous site; Ka: number of non-synonymous changes per non-synonymous site; π: nucleotide diversity.

**Figure 1:**
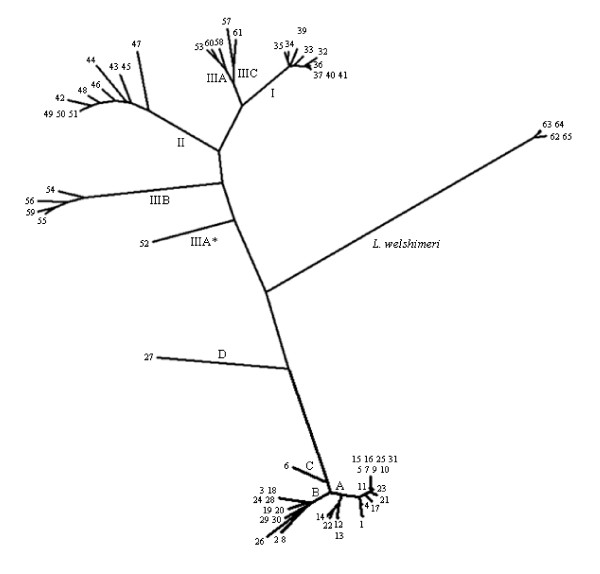
**Neighbor-joining cladogram of 34 *L. innocua *and 30 *L. monocytogenes *strains by the concatenated data set *gyrB-dapE-hisJ-sigB-ribC-purM-betL-gap-tuf *with *L. welshimeri *as outgroup species**. Leaves are labeled with sequence type (ST) designations. The numbers I, II, IIIA, IIIB, IIIC, A, B, C and D, on the branches represent *L. monocytogenes *lineages I, II, IIIA, IIIB, and IIIC, and *L. innocua *subgroups A, B, C and D respectively. IIIA* represents the low-virulent *L. monocytogenes *sublineages IIIA (seovar 4a) strain [11].

With *L. welshimeri *as the outgroup species, the phylogenetic tree revealed nine major branches of the *L. monocytogenes*-*L. innocua *clade, four corresponding to the recognized *L. monocytogenes *lineages I, II, IIIA/C and IIIB, one harboring the low-virulent *L. monocytogenes *lineage IIIA strains reported in our previous study [11], and the other four beloning to *L. innocua *(Figure 1). The majority of *L. innocua *strains were placed in two branches: one contained 19 strains (55.9%) representing STs 1, 4, 5, 7, 9-17, 21-23, 25 and 31, and the other harbored 13 strains (38.2%) representing STs 2, 3, 6, 8, 18-20, 24, 26 and 28-30. Remarkably, *L. innocua *strain L43 (ST27) showed the least genetic distance to the main cluster of *L. monocytogenes*. This strain seems to serve as the evolutionary intermediate between *L. monocytogenes *and *L. innocua *main clusters together with the low-virulent *L. monocytogenes *lineage IIIA strain 54006. Additionally, *L. innocua *strain 0063 (ST6) was present on the halfway between the *L. innocua *main cluster and strain L43 (Figure 1).

Based on the MLST scheme and internalin profiling, *L. innocua *could be divided into at least four subgroups. Two main subgroups A and B located in the two major branches of the phylogenetic tree, which correlated with IT1 and IT3 (except strain 0063) (19/34, 55.9%), and IT2 and IT4 (13/34, 38.2%) respectively. In addition, one IT3 strain 0063 and one IT5 strain L43 present in two individual branches formed subgroups C and D respectively (Table 2).

### Phylogeny and population history of *L. innocua*

As aforementioned, *L. innocua *was genetically monophyletic (π = 1.06%) as compared to *L. monocytogenes *(π = 4.38%). When sequence data were analyzed after stratification by subgroups, the number of polymorphisms and genetic diversity within each subpopulations were reduced (Table 3), suggesting a barrier for genetic exchange between these *L. innocua *subgroups. Such barrier was also observed between *L. monocytogenes *lineages (Table 3), consistent with one previous report [21].

Tajima's D test revealed that *L. innocua *and *L. monocytogenes *did not evolve under neutrality. A marginal positive value of Tajima's D observed for *ribC *in *L. monocytogenes *(1.9963, 0.05 < p < 0.10) became smaller or negative when analyses were performed for separate lineages, suggesting a divided population structure. Similarly, significant or marginal positive Tajima's D values were observed for *gyrB *(2.0401, p < 0.05) in *L. monocytogenes *lineage II, and for *sigB *(2.0426, p < 0.05) and *gap *(1.7746, 0.05 < p < 0.10) in lineage III, supporting that lineages II and III represented diverse populations as compared to lineage I (Table 4). On the other hand, *gyrB *(-2.2650, p < 0.01), *betL *(-2.5954, p < 0.001) and *gap *(-2.4190, p < 0.01) showed significant negative Tajima's D values in *L. innocua*, indicative of a bottleneck or selective sweep [22,23]. Also, Tajima's D were marginal negative for *betL *in *L. innocua *subgroup A (-1.7315, 0.05 < p < 0.10) and *gap *in subgroup B (-1.6523, 0.05 < p < 0.10) (Table 4).

**Table 4:** Tajima's D test for the *L. innocua*-*L. monocytogenes *clade

Gene	*L. innocua*			*L. monocytogenes*			
	
	A	B	all	I	II	III	all
*gyrB*	-0.3479	0.3871	-2.2650**	-1.6671#	2.0401*	0.0136	0.7361
*dapE*	0.7970	1.1138	-1.0723	-0.0394	-0.4958	0.9003	-0.3265
*hisJ*	1.2046	0.1750	0.2478	-0.1104	-0.6528	0.0336	1.4256
*sigB*	-0.1097	0.5901	0.2092	0.5444	-1.1117	2.0426*	1.2456
*ribC*	0.0511	0.2773	0.2987	1.5368	-1.5344	0.4909	1.9963#
*purM*	0.5044	0.2217	-1.4464	0.0235	-0.2856	0.9867	0.4698
*betL*	-1.7315#	-1.5047	-2.5954***	-0.2912	-0.1839	0.5179	0.0554
*gap*	-1.1648	-1.6523#	-2.4190**	-0.6910	-0.8223	1.7746#	0.2481
*tuf*	N/A^a^	0.9505	-0.0101	N/A	0.8198	0.5380	0.4709
Concatenated	0.1719	0.1492	0.3847	0.3655	-0.7070	0. 7379	0.7452

#, 0.05 < p < 0.10; *, p < 0.05; **, p < 0.01; ***, p < 0.001.

a. No polymorphisms in the data, resulting in inability to compute Tajima's test.

**Figure 2:**
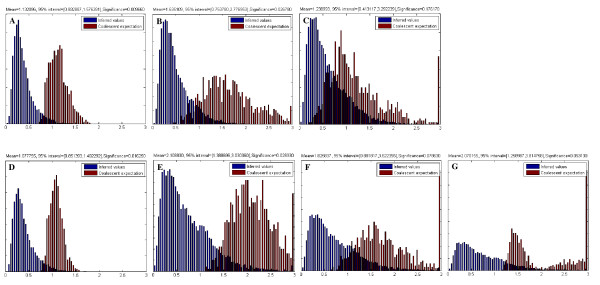
**Population history in *L. innocua*-*L. monocytogenes *clade inferred by the distribution of the exterior/interior branch length ratio of trees resulting from ClonalFrame analysis as compared to trees simulated under the coalescent model**. *L. innocua *spp. (A) and its group subgroup I (B), and *L. monocytogenes *spp. (D) and its lineage I (E) show a significantly smaller exterior/interior branch length ratio (p < 0.05) than expected under the coalescent model, while *L. innocua *subgroup II (C) and *L. monocytogenes *lineages II (F) and III (G) do not.

The exterior/interior branch length ratio test demonstrated that *L. innocua *and its subgroup A as well as *L. monocytogenes *and its lineage I showed a significantly smaller exterior/interior branch length ratio (p < 0.05) than expected under the coalescent model (Figure 2). This suggests that the contemporary *L. innocua *population experienced a recent expansion of its population size, consistent with a population bottleneck. Specifically, *L. innocua *subgroup A underwent expansion of the population size (p = 0.027), while subgroup II did not (p = 0.176) (Figure 2).

The rate of recombination within bacterical species can differ widely from one species to another. In the *L. innocua*-*L. monocytogenes *clade, both the relative frequency of occurrence of recombination versus mutation (ρ/θ) and the relative effect of recombination versus point mutation (r/m) were about two to three times higher in *L. innocua *than in *L. monocytogenes *(Table 5). *L. innocua *subgroup A exhibited significantly higher frequency (ρ/θ = 3.7697) and effect (r/m = 12.0359) of recombination than subgroup B (ρ/θ = 0.2818; r/m = 4.8132), consistent with a definite population expansion of subgroup A as aforementioned. However, the higher recombination rate of *L. innocua *subgroup A did not seem to contribute to nucleotide diversity (π for subgroups A and B are 0.46% and 0.77% respectively) (Table 3 and Table 5). On the other hand, both the frequency and effect of recombination in *L. monocytogenes *lineage II were higher than those in lineages I and III (Table 5).

**Table 5:** Recombination rates in the *L. innocua*-*L. monocytogenes *clade and other bacteria

	r/m^a^	ρ/θ^b^	Reference
*L. innocua*	3.144 (2.234-4.071)	0.535 (0.396-0.764)	This study
*L. innocua *subgroup A	12.036 (5.404-20.716)	3.770 (2.021-6.188)	This study
*L. innocua *subgroup B	4.813 (1.431-20.455)	0.282 (0.095-1.124)	This study
*L. monocytogenes*	1.847 (1.293-2.641)	0.179 (0.135-0.258)	This study
*L. monocytogenes *lineage I	5.752 (1.413-18.660)	0.055 (0.023-0.118)	This study
*L. monocytogenes *lineage II	7.610 (5.096-11.065)	0.518 (0.244-0.801)	This study
*L. monocytogenes *lineage III	1.869 (0.720-5.117)	0.195 (0.066-0.661)	This study
*L. innocua*-*L. monocytogenes *clade	2.783 (2.326-3.307)	0.334 (0.284-0.395)	This study
*Bacillus anthracis-Bacillus cereus *clade	ND	0.2-0.5	Didelot *et al. *2007
*Clostridium perfringens*	ND	3.2	Rooney *et al. *2006
*Neisseria meningitis*	ND	1.1	Jolley *et al. *2005
*Staphylococcus aureus*	ND	0.11	Fraser *et al. *2005
*Streptococcus pneumoniae*	ND	2.1	Fraser *et al. *2005

ND, not done.

a. The ratio of probabilities that a given site is altered through recombination and mutation, representing a measure of how important the effect of recombination is in the diversification of the sample relative to mutation.

b. The ratio of rates at which recombination and mutation occur, representing a measure of how often recombination events happen relative to mutations.

**Figure 3:**
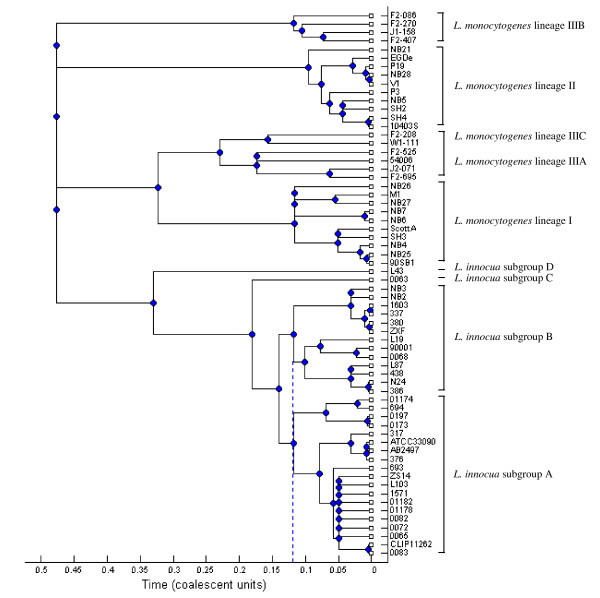
**A 95% majority-rule consensus tree based on ClonalFrame output with correction for recombination**. The X-axis represents the estimated time to the most recent common ancestors (TMRCA) of the *L. innocua*-*L. monocytogenes *clade. Blue dash line shows the estimated time to the most recent common ancestors of *L. innocua *subgroups I and II.

The phylogram based on the analysis with correction for recombination revealed that the time to the most recent common ancestor (TMRCA) of *L. innocua *subgroups A and B was similar (Figure 3), suggesting that these two subgroups appeared at approximately the same time. In addition, our study also showed the TMRCA of *L. monocytogenes *lineages I and II were similar, consistent with a recent report [24].

### Distribution of *L. innocua *isolates among different sources

Of the 29 *L. innocua *food isolates, 13 were obtained from meat, 8 from milk and 8 from seafoods. The majority of meat isolates (10/13, 76.9%) belonged to subgroup A, while most seafood isolates (5/8, 62.5%) belonged to subgroup B. There were significant associations between subgroups and source of isolation (p < 0.05).

### *L. innocua *isolates lack virulence genes found in *L. monocytogenes*, and were nonpathogenic to mice

All *L. innocua *strains lacked 17 virulence genes examined, with the exception of the subgroup D strain (L43) harboring *inlJ *(87.5%-93.6% nucleotide identities to *L. monocytogenes *reference strains EGDe and F2365) and two subgroup B strains (1603 and 386) bearing *bsh *(97.7%-99.4% nucleotide identities to EGDe and F2365). All of these *L. innocua *strains were nonpathogenic to ICR mice (Table 1).

## Discussion

The ecological, biochemical and genetical resemblance as well as the clear differences of virulence between *L. monocytogenes *and *L. innocua *make this bacterial clade attractive as models to examine the evolution of pathogenicity in *Listeria *genus. *L. monocytogenes *causes life-threatening infections in animals and human populations, and exhibits a diversity of strains with different pathogenicity [25]. *L. innocua *has once been postulated as the nonpathogenic variant of *L. monocytogenes*, and holds the key to understanding the evolutionary history of the *L. monocytogenes*-*L. innocua *clade. However, information on the phylogenetic structure and microevolution of *L. innocua *is still lacking. Thus, we characterized *L. innocua *strains in our laboratory stock from phylogentic perspectives.

Profiling of 37 internalin genes grouped the *L. innocua *strains into five internalin types, IT1 to IT5, with IT1 and IT2 as the major types (Table 2). The MLST scheme identified two major phylogenetic branches containing the majority of sequence types (29/31, 93.5%), and other two bearing one strain each (Fig 1). Consequently, *L. innocua *consists of at least four distinct subgroups A, B, C and D. Subgroup A correlates with one of the major branches including all the IT1 and IT3 strains with the exception of one IT3 strain 0063 belonging to subgroup C, while subgroup B correlates with the other major branch covering all the IT2 and IT4 strains (Table 2B). Therefore, it is inferred that a certain *L. innocua *subgroup possibly contains several serovars and exhibits different internalin patterns, which is similar to the fact that each lineage of *L. monocytogenes *contains several serovars and exhibits more than one internalin patterns, as exemplified by the internalin island between *ascB *and *dapE *in our previous report [17].

The majority of *L. monocytogenes *lineage I strains harbor *inlC2DE*, and a small number of 1/2b strains carry *inlGC2DE *instead. Within *L. monocytogenes *lineage II strains, the majority of 1/2a and 1/2c strains harbor *inlGC2DE *and *inlGHE *respectively. In addition, *L. monocytogenes *lineage III strains show the greatest level of diversity [8,17]. The *L. innocua *subgroup A strains either contain a whole set of *L. monocytogenes*-*L. innocua *common and *L. innocua*-specific internalin genes, or lack *lin1204 *and *lin2539*, and the *L. innocua *subgroup B strains either lack *lin1204 *or lack *lin0661*, *lin0354 *and *lin2539 *instead. Besides, the subgroup D strain L43, which shows the least genetic distance to *L. monocytogenes*, lacks *lin1204 *but bears *L. monocytogenes*-specific *inlJ *in the counterpart region in *L. monocytogenes *genomes (Table 2). We propose that certain internalin genes such as *lin0354*, *lin0661*, *lin1204 *and *lin2539 *could be potential genetic markers for subgroups of *L. innocua*.

The phylogenetic tree revealed nine major branches of the *L. innocua*-*L. monocytogenes *clade, five belonged to *L. monocytogenes *representing lineages I, II, and III, consistent with previous reports [11,24,26], and the other four represented *L. innocua *subgroups A, B, C and D (Fig 1). Overall, *L. innocua *is genetically monophyletic compared to *L. monocytogenes*, and the nucleotide diversity of the *L. innocua *species is similar to that of *L. monocytogenes *lineage I but less than those of *L. monocytogenes *lineages II and III. In evolutionary terms, younger bacterial species has lower level of genetic diversity [15]. The results from this study offer additional evidence that *L. innocua *possibly represents a relatively young species as compared to its closest related pathogenic species *L. monocytogenes*.

Previous studies suggest that *L. monocytogenes *represents one of the bacterial species with the lowest rate of recombination [4,27]. In this study, strains in the *L. innocua*-*L. monocytogenes *clade exhibit similar value of ρ/θ to those of the *Bacillus anthracis-Bacillus cereus *clade [28] and slightly higher than those of *Staphylococcus aureus *[29], but still considerably lower than those of pathogens such as *Clostridium perfringens *[30], *Neisseria meningitis *[31] and *Streptococcus pneumoniae *[29]. Both the relative frequency of occurrence of recombination versus mutation (ρ/θ) and the relative effect of recombination versus point mutation (r/m) of *L. innocua *were higher than those of *L. monocytogenes*. More strikingly, recombination rates of *L. innocua *subgroup A were particularly high (Table 5). Wirth et al. [32] proposed from the data for *Escherichia coli *that epidemic and virulent bacteria face an increased selective pressure for rapid diversification in response to host immune defenses, resulting in higher recombination rates. *L. monocytogenes *is an opportunistic pathogen with wide host ranges as well as a saprotroph found in different environments [2,33]. Though lineage I strains were responsible for almost all major human listeriosis outbreaks and the majority of sporadic cases [6], those of lineage II exhibited higher recombination rate according to our observation and the findings by Bakker et al. [24]. Bakker et al. [24] proposed that higher recombination in lineage II was not due to selective forces involved in its virulence. Recombination may be critical for lineage II to successfully compete and survive in a board range of different environments. Lineage II strains are more commonly found at higher levels than lineage I strains in natural environments including foods [24,34]. Similarly, we postulate that the nonpathogenic species *L. innocua *descending from its pathogenic ancestor has better adaptability to contemporary environmental niches. Removal of some gene loci related to virulence (e.g., LIPI-1, *inlAB *and *bsh*) in *Listeria *could be regarded as adaptive gene loss, which favors its survival in environmental niches as a saprotroph [9,11].

*L. innocua *subgroups A and B strains have similar TMRCA and exhibit similar genetic distances to *L. monocytogenes*, suggesting that these two subgroups appeared at approximately the same time (Fig 2). However, subgroup A experienced a recent expansion of the population size, consistent with the higher recombination frequency (r/m) and effect (ρ/θ) of subgroup A as compared to those of subgroup B. This further implies that these two subgroups have distinct inclinations and adaptive abilities to environments and occupy different habitats, while subgroup A might face increased selective pressures resulting in higher recombination rates. Additional support for this indication is that the majority of subgroup A isolates (belonging IT1) contain a whole set of *L. monocytogenes*-*L. innocua *common and *L. innocua*-specific internalin genes which may play broad roles in enhancing the adaption to various environments. Hence, the *L. innocua *subgroup A strains might represent the possible evolutionary direction towards adaptation. Interestingly, the higher recombination rate of *L. innocua *subgroup A did not seem to contribute to nucleotide diversity. One possible explanation is that members of subgroup A recombines only with other members of subgroup A, so that recombination does not introduce novel polymorphisms into subgroup A and therefore does not increase the genetic diversity.

Previous reports indicate that horizontal gene transfer might have occurred earlier to form a more ancestral *L. monocytogenes *strain, which would then give rise to *L. innocua *through gene deletion events possibly via low-virulent *L. monocytogenes *lineage IIIA strains [11,13]. In this study, *L. innocua *subgroup D strain L43 exhibits the least genetic distances to *L. monocytogenes *(Fig 1), and constituted another evolutionary intermediates between *L. monocytogenes *and *L. innocua *main clusters. Therefore, *L. innocua *strain L43 and *L monocytogenes *strain 54006 [11] might serve as intermediate linkage strains in deciphering the evolution of the *L. innocua*-*L. monocytogenes *clade.

The strain L43 seems to share a "hybrid" genetic background derived from *L. innocua *and *L. monocytogenes *by the MLST data and its carriage of *L. monocytogenes*-specific virulence gene *inlJ*. InlJ is a sortase-anchored adhesin specifically expressed *in vivo *[35], but its function in atypical *L. innocua *strains requires further investigation. Another atypical *L. innocua *strain PRL/NW 15B95 has been characterized as having the entire LIPI-1 embedded into an otherwise typical *L. innocua *genetic background [9]. However, we did not see its presence in the strain L43. PRL/NW 15B95 falls into the main *L. innocua *cluster based on sequencing of 16S-23S intergenic regions, 16S rRNA and *iap *genes, and has possibly acquired LIPI-1 by a later transposition event, based on the finding of a 16 bp Tn1545 integration consensus sequence flanking the virulence island [9]. Thus, unlike L43, PRL/NW 15B95 does not constitute an evolutional intermediate between *L. monocytogenes *and *L. innocua*. Complementary transfer of only some of the virulence genes such as LIPI-1 did not change the avirulent character of PRL/NW 15B95 [9]. In this study, all *L. innocua *strains were nonpathogenic in mice models (Table 1).

## Conclusion

This study reveals that *L. innocua *is a relatively young species descending from *L. monocytogenes*. The evolutionary history in the *L. monocytogenes*-*L. innocua *clade represents a rare example of evolution towards reduced virulence of pathogens. *L. innocua *is genetically monophyletic and comprises four subgroups based on internalin profiling and MLST scheme. The majority of *L. innocua *strains belong to two major subgroups A and B, and one atypical subgroup might serve as a link between *L. monocytogenes *and *L. innocua *main cluster in the evolutionary chain. While subgroups A and B appeared at approximately the same time, the subgroup A strains seem to represent the possible evolutionary direction towards adaptation to enviroments. It is believed that the phylogenetic structure and evolutionary history of *L. innocua *will be much clearer if a larger strain collection and the whole genome sequences of more representative strains become available.

## Methods

### Bacterial strains

A total of 68 *Listeria *strains were examined in this study (Table 1). These included 30 *L. monocytogenes *strains representing three lineages, 34 *L. innocua *strains, 5 from reference collections, 13 from meat, 8 from milk and 8 from seafoods, and 4 *L. welshimeri *strains. *Listeria *strains were retrieved from glycerol stocks maintained at -80°C, and cultured in brain heart infusion broth (BHI; Oxoid, Hampshire, England) at 37°C.

### Carbohydrate fermentation and hemolytic reactions

The recommended biochemical patterns for differentiating *Listeria *spp. included L-rhamnose, D-xylose, D-mannitol and glucose utilization and hemolytic reactivity, and were tested by using conventional procedures [36,37].

### DNA manipulations

Genomic DNA was extracted using a protocol reported previously [12]. Oligonucleotide primers were synthesized by Invitrogen Biotechnology (Shanghai, China) (Table 6 and Additional file 1; table S2), and *Taq *DNA polymerase (TaKaRa Biotech Co. Ltd., Dalian, China) was used for PCR amplification. PCR was conducted using a PT-200 thermal cycler (MJ Research Inc. MA, Boston, USA), with annealing temperatures depending on specific primer pairs (Table 6 and Additional file 1; table S2), and the duration of extension depending on the expected length of amplicon (1 min per kb, at 72°C). For DNA sequencing analysis, PCR fragments were purified with the AxyPrep DNA Gel Extraction Kit (Axygen Inc., USA) and their sequences determined by dideoxy method on ABI-PRISM 377 DNA sequencer.

**Table 6:** Primers used for MLST

Locus	Putative function	Location^a^	Forward primer	Reverse primer	Length (bp)
*gyrB*	DNA gyrase subunit B	6,031-7,971	TGGTGCATCGGTAGTTAATGC	CAACATCTGGGTTTTCCATCAT	657
*dapE*	Succinyl diaminopimelate desuccinylase	301,402-302,538	GTAAATATTGATTCGACTAATG	CACTAGCACTTGTTTCACTG	669
*hisJ*	Histidinol phosphate phosphatase	606,408-607,235	TCCACATGGTACGCATGAT	GGACATGTCAAAATGAAAGATC	714
*sigB*	Stess responsive alternative sigma factor B	924,734-925,513	CCAAAAGTATCTCAACCTGAT	CATGCATTTGTGATATATCGA	642
*ribC*	Riboflavin kinaseand FAD synthase	1,364,536-1,365,480	AAGACGATATACTTACATCAT	GTCTTTTTCTAACTGAGCA	633
*purM*	Phosphoribosyl aminoimidazole synthase	1,893,107-1,894,153	CAAGCTCCACTTTGACAGCTAA	TAAAGCAGGCGTGGACGTA	693
*betL*	Glycine betaine transporter	2,216,882-2,218,405	ACAGAACATTATCCAAATGAGTT	ACGTTGTGATTTTTTCGGTC	534
*gap*	Glyceraldehyde 3-phosphate dehydrogenase	2,578,558-2,579,584	CTGGATCAGAAGCTGCTTCCA	GTCGTATTCAAAATGTGGAAGGA	621
*tuf*	Translation elongation factor	2,816,958-2,818,145	CATTTCTACTCCAGTTACTACT	GCTCTAAACCCCATGTTA	681
Subtotal					5,844

a, Positions correspond to complete genome sequence of *L. innocua *strain CLIP11262 (AL592022).

### Internalin profiling

By sequence comparison of *L. monocytogenes *strains F2365, H7858 (serovar 4b), EGDe and F6854 (serovar 1/2a) and *L. innocua *strain CLIP11262, we investigated the presence or absence of 14 *L. monocytogenes-L. innocua*-common and 4 *L. innocua*-specific internalin genes as well as 19 *L. monocytogenes*-specific internalin genes by PCR with specific primers outlined in Additional file 1; table S1. Due to the conserved repeats present in internalin multigene family [19], primers were designed based on the distinguishable regions through sequence comparison. As *inlH *and *inlC2 *shared highly identical nucleotide sequences, a common primer set was employed [17].

### Multilocus sequence typing (MLST)

The MLST scheme was based on the sequence analysis of 9 unlinked genes, including 7 housekeeping genes *gyrB*, *dapE*, *hisJ*, *ribC*, *purM*, *gap *and *tuf*, and 2 stress-response genes *sigB *and *betL*. Sequences generated in this study have been deposited in GenBank within the accession numbers FJ774089 to FJ774121 (*gyrB*), FJ774145 to FJ774177 (*sigB*), FJ774274 to FJ774282, FJ774257 to FJ774273, FJ774283 to FJ774293, FJ774295 to FJ774297, FJ774299 to FJ4300 (*gap*), FJ774313 to FJ774344, FJ774368 (*hisJ*), FJ774369 to FJ774400, FJ774424 (*purM*), FJ774425 to FJ774457 (*ribC*), FJ774481 to FJ774513 (*dapE*), FJ774537 to FJ774568 (*tuf*), and FJ774593 to FJ774625 (*betL*).

### Detection of virulence genes

Five categories of virulence genes found in *L. monocytogenes *were assessed by using primers listed in Additional file 1; table S2, including (i) stress response genes conferring tolerance to harsh conditions within the host (e.g. *bsh*, *arcB*, *arcD*, *lmo0038 *and *arcC*); (ii) internalin genes responsible for adhesion and invasion of host cells (e.g. *inlA*, *inlB*, *inlC*, *inlF *and *inlJ*); (iii) genes involved in escape from vacuole and intracellular multiplication (e.g. *plcA*, *hly*, *mpl*, *plcB *and *hpt*); (iv) the gene associated with intracellular and intercellular spread (e.g. *actA*); and (v) regulatory genes (e.g. *prfA*).

### Mouse infection

The virulence potential of 33 *L. innocua *strains and 30 *L. monocytogenes *isolates was assessed in ICR mice by a previously reported protocol [38]. The animal experiment was approved by the Laboratory Animal Management Committee of Zhejiang University, and the mice were handled under strict ethical conditions. Briefly, 5 female ICR mice at 20-22 g (Zhejiang College of Traditional Chinese Medicine, Hangzhou, China) were inoculated intraperitoneally with ~10^8 ^CFU each strain in a 0.1 ml-volume. Mice in the control group were injected with 0.1 ml PBS. The mice were observed daily and mortalities recorded until all of the mice inoculated with the virulent EGDe strain died. Relative virulence (%) was calculated by dividing the number of dead mice with the total number of mice tested. On the 15th day post- inoculation, all surviving mice were euthanized.

### Data analysis

For each MLST locus, an allele number was given to each distinct sequence variant, and a distinct sequence type (ST) number was given to each distinct combination of alleles of the 9 genes. MEGA 4.0 was used to construct a neighbor-joining tree of *L. innocua *and *L. monocytogenes *isolates using the number of nucleotide differences in the concatenated sequences of 9 loci with 1,000 bootstrap tests [39]. *L. welshimeri *was used as outgroup species. DNAsp v4.10.9 [40] was used to calculate the number of alleles, number of polymorphic sites, nucleotide diversity (π, mean pairwise nucleotide difference per site), Tajima's D (for testing the hypothesis that all mutations are selectively neutral [41]), numbers of synonymous mutations and nonsynonymous mutations and the rate of nonsynonymous to synonymous changes with a Jukes-Cantor correction. Discrimination index (D.I.) values of selected genes were calculated according to the method previously described [42] on the basis of allelic types [j], numbers of strains belonging to each type [nj], and the total numbers of strains analyzed [N] with the following equation (higher D.I. values indicate better discriminatory power):

ClonalFrame v1.1 was employed to show the evolution of ρ/θ and r/m as chain run. These two complementary measures were used to assess the relative contribution of recombination and mutation in the creation of the sample population from a common ancestor. Specifically, ρ/θ is the ratio of rates at which recombination and mutation occur, representing a measure of how often recombination happen relative to mutation [43], while r/m is the ratio of probabilities that a given site is altered through recombination and mutation, representing a measure of how important the effect of recombination is in the diversification of the sample population relative to mutation [44].

To infer the population history in the *L. innocua*-*L. monocytogenes *clade, ClonalFrame GUI, which treated each gene as an independent unit in the input file, was used to calculate the ratio of the sum of the external branches (the ones that connect a leaf of the tree) to the sum of the internal branches (the ones that connect two internal nodes of the tree) [45]. The distribution of these ratios was then compared to the distribution of the external/internal branch length ratio as expected under the coalescent model [46]. If the external/internal branch length ratio is significantly smaller than expected, it means that the inferred genealogy is unexpectedly "star-like", which is consistent with an expansion of the population size.

The chi-squared test was used to test significant associations between *L. innocua *subgroup and isolate source.

## Authors' contributions

JC conceived and designed the study, performed and interpreted the phylogenetic and statistical analyses, participated in the collection of the sequence data and animal assays, and drafted the manuscript. QC performed the PCR amplification and participated in the collection of the sequence data. LJ participated in evaluation of the results and in revision of the manuscript. CC and FB participated in the PCR amplification, biochemical tests and animal assays. JW and FM participated in the analysis of sequence data. WF supervised the project, participated in the design of the study and data interpretation, and helped draft the manuscript. All authors read and approved the final manuscript.

## Supplementary Material

Additional file 1 Table S1 and S2: Internalin Types (ITs) based on 14 *L. monocytogenes-L. innocua*-common and 4 *L. innocua*-specific (in grey shading) internalin genes. Click here for file
