# Supplementary material for: Internalin profiling and multilocus sequence typing suggest four Listeria innocua subgroups with different evolutionary distances from Listeria monocytogenes
Source: BMC Microbiol. 2010 Mar 31;10:97. doi: 10.1186/1471-2180-10-97 (PMC2867954; doi:10.1186/1471-2180-10-97)
Supplement: Additional file 1 — Table S1 and S2: Internalin Types (ITs) based on 14 L. monocytogenes-L. innocua-common and 4 L. innocua-specific (in grey shading) internalin genes. [file 1471-2180-10-97-S1.DOC]

**Additional files**

| Strain | *lin0295* | *lin0352* | *lin0354* | *lin0372* | *lin0514* | *lin0553* | *lin0619* | *lin0741* | *lin1101* | *lin1204* | *lin1328* | *lin2495* | *lin2537* | *lin2539* | *lin0558* | *lin0661* | *lin0739* | *lin2724* | No. | IT |
| --- | --- | --- | --- | --- | --- | --- | --- | --- | --- | --- | --- | --- | --- | --- | --- | --- | --- | --- | --- | --- |
| *L. innocua* |  |  |  |  |  |  |  |  |  |  |  |  |  |  |  |  |  |  |  |  |
| ATCC33090 | + | + | + | + | + | + | + | + | + | + | + | + | + | + | + | + | + | + | 18 | 1 |
| 90001 | + | + | + | + | + | + | + | + | + | – | + | + | + | + | + | + | + | + | 17 | 2 |
| 1603 | + | + | + | + | + | + | + | + | + | – | + | + | + | + | + | + | + | + | 17 | 2 |
| AB2497 | + | + | + | + | + | + | + | + | + | + | + | + | + | + | + | + | + | + | 18 | 1 |
| CLIP11262 | + | + | + | + | + | + | + | + | + | + | + | + | + | + | + | + | + | + | 18 | 1 |
| 0063 | + | + | + | + | + | + | + | + | + | – | + | + | + | – | + | + | + | + | 16 | 3 |
| 0065 | + | + | + | + | + | + | + | + | + | + | + | + | + | + | + | + | + | + | 18 | 1 |
| 0068 | + | + | + | + | + | + | + | + | + | – | + | + | + | + | + | + | + | + | 17 | 2 |
| 0072 | + | + | + | + | + | + | + | + | + | + | + | + | + | + | + | + | + | + | 18 | 1 |
| 0082 | + | + | + | + | + | + | + | + | + | + | + | + | + | + | + | + | + | + | 18 | 1 |
| 0083 | + | + | + | + | + | + | + | + | + | + | + | + | + | + | + | + | + | + | 18 | 1 |
| 0173 | + | + | + | + | + | + | + | + | + | – | + | + | + | – | + | + | + | + | 16 | 3 |
| 0197 | + | + | + | + | + | + | + | + | + | – | + | + | + | – | + | + | + | + | 16 | 3 |
| 01174 | + | + | + | + | + | + | + | + | + | + | + | + | + | + | + | + | + | + | 18 | 1 |
| 01178 | + | + | + | + | + | + | + | + | + | + | + | + | + | + | + | + | + | + | 18 | 1 |
| 01182 | + | + | + | + | + | + | + | + | + | + | + | + | + | + | + | + | + | + | 18 | 1 |
| 317 | + | + | + | + | + | + | + | + | + | + | + | + | + | + | + | + | + | + | 18 | 1 |
| 337 | + | + | + | + | + | + | + | + | + | – | + | + | + | + | + | + | + | + | 17 | 2 |
| 376 | + | + | + | + | + | + | + | + | + | + | + | + | + | + | + | + | + | + | 18 | 1 |
| 380 | + | + | + | + | + | + | + | + | + | – | + | + | + | + | + | + | + | + | 17 | 2 |
| 386 | + | + | + | + | + | + | + | + | + | – | + | + | + | + | + | + | + | + | 17 | 2 |
| 438 | + | + | + | + | + | + | + | + | + | – | + | + | + | + | + | + | + | + | 17 | 2 |
| 693 | + | + | + | + | + | + | + | + | + | + | + | + | + | + | + | + | + | + | 18 | 1 |
| 694 | + | + | + | + | + | + | + | + | + | + | + | + | + | + | + | + | + | + | 18 | 1 |
| ZS14 | + | + | + | + | + | + | + | + | + | + | + | + | + | + | + | + | + | + | 18 | 1 |
| ZXF | + | + | + | + | + | + | + | + | + | – | + | + | + | + | + | + | + | + | 17 | 2 |
| 1571 | + | + | + | + | + | + | + | + | + | + | + | + | + | + | + | + | + | + | 18 | 1 |
| L19 | + | + | – | + | + | + | + | + | + | + | + | + | + | – | + | – | + | + | 15 | 4 |
| L43 | + | + | + | + | + | + | + | + | + | – | + | + | + | + | + | + | + | + | 16 | 5 |
| NB2 | + | + | + | + | + | + | + | + | + | – | + | + | + | + | + | + | + | + | 17 | 2 |
| NB3 | + | + | + | + | + | + | + | + | + | – | + | + | + | + | + | + | + | + | 17 | 2 |
| NB24 | + | + | + | + | + | + | + | + | + | – | + | + | + | + | + | + | + | + | 17 | 2 |
| L87 | + | + | + | + | + | + | + | + | + | – | + | + | + | + | + | + | + | + | 17 | 2 |
| L103 | + | + | + | + | + | + | + | + | + | + | + | + | + | + | + | + | + | + | 18 | 1 |
| *L. monocytogenes* |  |  |  |  |  |  |  |  |  |  |  |  |  |  |  |  |  |  |  |  |
| ScottA | + | + | + | + | + | + | + | + | + | + | – | + | + | + | – | – | – | – | 13 | – |
| EGDe | – | + | + | – | + | + | + | + | + | – | + | + | – | + | – | – | – | – | 10 | – |
| 54006 | + | + | + | + | + | + | + | + | – | – | – | + | + | + | – | – | – | – | 11 | – |
| F2-695 | + | + | + | + | + | + | + | + | – | – | – | + | + | + | – | – | – | – | 11 | – |
| F2-086 | – | – | + | + | + | – | + | + | – | – | – | + | – | – | – | – | – | – | 6 | – |
| F2-407 | – | – | + | + | + | – | + | + | – | – | – | + | – | – | – | – | – | – | 6 | – |
| F2-270 | – | – | + | + | + | – | + | + | – | – | – | + | – | – | – | – | – | – | 6 | – |

**Additional Table1 Internalin Types (ITs) based on 14 *L. monocytogenes-L. innocua*-common and 4 *L. innocua*-specific (in grey shading) internalin genes**

| F2-208 | + | + | + | + | + | + | + | + | – | – | – | + | + | + | – | – | – | – | 11 | – |
| --- | --- | --- | --- | --- | --- | --- | --- | --- | --- | --- | --- | --- | --- | --- | --- | --- | --- | --- | --- | --- |
| F2-525 | + | + | + | + | + | + | + | + | – | – | – | + | + | + | – | – | – | – | 11 | – |
| J1-158 | – | + | + | + | + | – | + | + | – | – | – | + | – | – | – | – | – | – | 7 | – |
| J2-071 | + | + | + | + | + | + | + | + | – | – | – | + | + | + | – | – | – | – | 11 | – |
| W1-111 | + | + | + | + | + | – | + | + | – | – | – | + | + | + | – | – | – | – | 10 | – |

**Additional Table 2 Primers used for internalin profiling and virulence genes analysis**

| Locus | Putative function | Forward primer | Reverse primer | Length (bp) | Annealing temperature (^o^C) | Reference |
| --- | --- | --- | --- | --- | --- | --- |
| *bsh* | Bile salt hydrolase | CCCACGACTATAAGCATCCA | GCAGGACTCAATTTCTCAGGA | 399 | 58 | This study |
| *arcB* | Catabolic ornithine carbamoyltransferase | ATGAGTGATTACAACCATCCA | GCTTCATCAATAACAACTGAG | 512 | 60 | This study |
| *arcD* | Arginine:ornithine antiporter | GTTGTTATCCAGTGAGCGATA | AATTTGCTACTAATGTATACA | 459 | 60 | This study |
| *lmo0038* | Putative stress response gene | TTGAAAAACACGCTGGTTGCT | CCAACCACTTCACAGTTTGGA | 967 | 62 | Chen et al., 2009 |
| *arcC* | Carbamate kinase | GCAATCTTTCTTGAGGATGCT | TGGGCAACATACTTCCTGCTG | 379 | 60 | This study |
| *prfA* | Transcriptional regulator | CCATACACATAGGTCAGGATT | TTCGTTATAATGTCTGGCTTT | 266 | 60 | This study |
| *plcA* | Phosphatidylinositol- phospholipase C | ATTAACCAAACCACTGGCTCA | TTGATAAGCAGTCTGGACAAT | 502 | 55 | This study |
| *hly* | Listeriolysin O | GTTGCAAGCGCTTGGAGTGAA | ACGTATCCTCCAGAGTGATGG | 420 | 58 | This study |
| *mpl* | Metalloenzyme | CAAGGACAGCTTAGGATTAC | TTCTTATTCGCCCATCTCGC | 886 | 55 | This study |
| *actA* | Actin-assembly inducing protein precursor | GGTACGTGATAAAATCGACGA | TAGTTATGTCACTTATCAGAGC | 537 or 432 | 55 | Wiedmann et al., 1997 |
| *plcB* | Phosphatidylcholine- phospholipase C | ATTAACCAAACCACTGGCTCA | TTGATAAGCAGTCTGGACAAT | 502 | 55 | This study |
| *hpt* | Hexose phosphate transport | GATTTGTGCAATCACCAGGT | GAACCTAGCAATGCTCCAAT | 529 | 58 | This study |
| *inlA* | Internalin A | TAATATAAGTGATATAAGCCCAG | TTTATCCGTACTGAAATTCC | 606 | 60 | Chen et al., 2009 |
| *inlB* | Internalin B | CACTTTCTTTGGAGCATAATGGT | CATCATCACTTATTATTTCTGGA | 394 | 60 | Chen et al., 2009 |
| *inlC* | Internalin C | CCATCTGGGTCTTTGACAGTA | CAAATAAGTGACCTTAGTCCTT | 398 | 55 | Chen et al., 2009 |
| *inlD* | Internalin D | CTGTAGTAATGGCAATTAGCTT | TGTTATTAGGGACCACAAGCT | 870 | 52 | Chen et al., 2009 |
| *inlE* | Internalin E | AGCTCAAAAGAAGTACAAGCA | GTGCAATAAGCTCACCAGAAA | 787 | 55 | Chen et al., 2009 |
| *inlF* | Internalin F | TGACTTATTTGCAGTTGGGGT | TTGGTTCAGGAATAAGCGCG | 1,119 | 55 | Chen et al., 2009 |
| *inlG* | Internalin G | GTGAAGACGGAACTTGGAAA | GCTTCTACTATCGGTTGAACA | 668 | 52 | Chen et al., 2009 |
| *inlH/C2* | Internalin H/ Internalin C2 | ATAGCTACTTTATCAGCATTT | ATATCACTTATTTTATTATCATC | 437 | 52 | Chen et al., 2009 |
| *inlI* | Internalin I | GTTTCCAGACGACAATCTTGCTA | AATCGGTACAGTTACTCGCATCA | 635 | 58 | Chen et al., 2009 |
| *inlJ* | Internalin J | AGATGTGACACCACAAACTCAA | TGTATTATGCGTGACATCAAGCT | 401 | 58 | Chen et al., 2009 |
| *lmo0171* | Internalin-like protein | ATTCAAGCAAACTTGGAACG | CTTCTGGTACTTTCACGGTGA | 351 | 55 | This study |
| *lmo0801* | Internalin-like protein | CTATCCAGAGGGAACAACAA | CTGTAAGAGGTAATTTGTCTCC | 428 | 58 | This study |
| *lmo1290* | Internalin-like protein | AATTATGATGGCTATGTACCAG | CAAAATCACTTGTAATCGCTG | 643 | 58 | This study |
| *lmo2026* | Internalin-like protein | CGGTTGTTCCTGATGTGTTGCTT | TCGACGAACTCTAATCCTTTTGC | 837 | 58 | This study |
| *lmo2027* | Internalin-like protein | GAACTCCGTTAGATCTTCTCCA | ATTAACCATAACTGGTGGAGATC | 295 | 55 | This study |
| *lmo2470* | Internalin-like protein | GATGGATTAGCCAAAGATATG | TTAGAGCGGATGTATCCACT | 934 | 58 | This study |
| *F0349* | Internalin-like protein | ATGGTTCATCAATTGAAGCTGA | CTCGGACTGACCTGTTGGAC | 683 | 55 | This study |
| *F0805* | Internalin-like protein | AAAAACAATTGCTCCCACTG | TTATAAGCCTCTCCCACACG | 681 | 55 | This study |
| *lin0295* | Internalin-like protein | TTGGAATGGTACTGAGAGGTG | TTCCCAGATGATGCTCTAGC | 613 | 55 | This study |
| *lin0352* | Internalin-like protein | GGCAAAGAACTACAAGCACCTA | TTGGTTCTTTGGTGCAGCTA | 531 | 58 | This study |
| *lin0354* | Internalin-like protein | CTTAAGCTAGTAATATCACCTGT | TACAGACCTGTTAGATAAGGAT | 600 | 55 | This study |
| *lin0372* | Internalin-like protein | TAACGGATGGTATGATGCTGA | AAAGTACACGATGATCTCCTGT | 656 | 58 | This study |
| *lin0514* | Internalin-like protein | GATCAGTATTTTGATATTAGCCA | TCAAAAATTCGTCGACTGAT | 331 | 58 | This study |
| *lin0553* | Internalin-like protein | AAGCTGGTGATTATGTTGTAA | ATTTACTTTGATACTTTAGCGA | 332 | 58 | This study |
| *lin0619* | Internalin-like protein | CGCTATCAAAATCACTAGTAAC | TTTTTGTACCATTTACTTTAATG | 444 | 58 | This study |
| *lin0741* | Internalin-like protein | TTGTTACTTCTGAAGATTTAGGA | TAATGTAAATCCAGCAATTTCT | 1175 | 55 | This study |
| *lin1101* | Internalin-like protein | AAAAGGTGTTCAAAGTCTTGA | AAAACATCAGCATATTAGACAG | 386 | 58 | This study |
| *lin1204* | Internalin-like protein | AAACATACACAACAGTGGCTA | CTTTCCCTGTTTCATCTACA | 387 | 58 | This study |
| *lin1328* | Internalin-like protein | TATTTCACAAGAAGGCGTTAC | AGAATCCTACTGCTACTCCAGT | 965 | 55 | This study |
| *lin2495* | Internalin-like protein | GATGGAGAAAAAATGCCTGTT | TTTGGTTAGCATTTGTAAATACG | 579 | 55 | This study |
| *lin2537* | Internalin-like protein | CCTGTAAATTTTTCAAGTTTGTA | CCAGCCAGAATAATTGATGT | 502 | 55 | This study |
| *lin2539* | Internalin-like protein | AGGCATATTCATCCAAATATCT | GCTGAAGAATTGGATAAAGATTC | 410 | 58 | This study |
| *lin0558* | Internalin-like protein | CAACTACTTGAGTACCTCGGT | TCCAGTTATAGTCCATCCTTG | 678 | 58 | This study |
| *lin0661* | Internalin-like protein | ACATACGAGATAATCAGATAGA | ATGTTGTTACTGTCTCCTGT | 880 | 55 | This study |
| *lni0739* | Internalin-like protein | TTCACTGGTTGGTACGATGCA | ATAGCCCAATCAGCACCAAGT | 337 | 58 | This study |
| *lin2724* | Internalin-like protein | GTTTGTGTCGTTGTTGTTCCTT | GAAGAACCAAATGGTTACGGA | 770 | 55 | This study |
